# Ciprofloxacin Modulates Cytokine/Chemokine Profile in Serum, Improves Bone Marrow Repopulation, and Limits Apoptosis and Autophagy in Ileum after Whole Body Ionizing Irradiation Combined with Skin-Wound Trauma

**DOI:** 10.1371/journal.pone.0058389

**Published:** 2013-03-08

**Authors:** Risaku Fukumoto, Lynnette H. Cary, Nikolai V. Gorbunov, Eric D. Lombardini, Thomas B. Elliott, Juliann G. Kiang

**Affiliations:** 1 Radiation Combined Injury Program, Uniformed Services University of the Health Sciences, Bethesda, Maryland, United States of America; 2 Radiation Countermeasures Program, Uniformed Services University of the Health Sciences, Bethesda, Maryland, United States of America; 3 Veterinary Sciences Department, Armed Forces Radiobiology Research Institute, Uniformed Services University of the Health Sciences, Bethesda, Maryland, United States of America; 4 Department of Radiation Biology, Uniformed Services University of the Health Sciences, Bethesda, Maryland, United States of America; 5 Department of Medicine, Uniformed Services University of the Health Sciences, Bethesda, Maryland, United States of America; University of Cincinnati, United States of America

## Abstract

Radiation combined injury (CI) is a radiation injury (RI) combined with other types of injury, which generally leads to greater mortality than RI alone. A spectrum of specific, time-dependent pathophysiological changes is associated with CI. Of these changes, the massive release of pro-inflammatory cytokines, severe hematopoietic and gastrointestinal losses and bacterial sepsis are important treatment targets to improve survival. Ciprofloxacin (CIP) is known to have immunomodulatory effect besides the antimicrobial activity. The present study reports that CIP ameliorated pathophysiological changes unique to CI that later led to major mortality. B6D2F1/J mice received CI on day 0, by RI followed by wound trauma, and were treated with CIP (90 mg/kg *p.o., q.d.* within 2 h after CI through day 10). At day 10, CIP treatment not only significantly reduced pro-inflammatory cytokine and chemokine concentrations, including interleukin-6 (IL-6) and KC (*i.e.*, IL-8 in human), but it also enhanced IL-3 production compared to vehicle-treated controls. Mice treated with CIP displayed a greater repopulation of bone marrow cells. CIP also limited CI-induced apoptosis and autophagy in ileal villi, systemic bacterial infection, and IgA production. CIP treatment led to LD_0/10_ compared to LD_20/10_ for vehicle-treated group after CI. Given the multiple beneficial activities of CIP shown in our experiments, CIP may prove to be a useful therapeutic drug for CI.

## Introduction

Nuclear disasters such as detonation of weapons could cause serious injury to a human body by a combination of radiation exposure and other insults that include physical wounds and thermal burns, namely radiation combined injury (CI). CI generally leads to greater mortality than radiation injury (RI) alone, even though each injury separately may not be lethal [1], [2], [3]. Development of countermeasures to CI is a pressing issue, and it requires knowledge in RI, in non-RI injury, and also in their combined effects.

We previously reported an experimental model for CI, in which wound trauma increased RI-induced cell death, tissue damage, organ dysfunction, and finally mortality in mice [3]. In B6D2F1/J female mice, the LD_50/30_ was 9.65 Gy for RI compared to only 8.95 Gy for CI, in which ^60^Co-γ-photons at 0.4 Gy/min and 15% total body surface area (TBSA) dorsal skin wound were applied. During the course of 30 days after CI, these mice also experienced greatly increased concentrations of pro-inflammatory cytokines and chemokines in serum [3], rapid loss in hematological parameters, including lymphocytes and granulocytes [4], gastrointestinal injury as well as systemic endogenous bacterial translocation from the intestinal tract and subsequent sepsis [3]. It is particularly important to manage these pathophysiological changes, in order to achieve enhanced survival.

Ciprofloxacin (CIP) is an FDA-approved fluoroquinolone (FQ), which is widely used as an antimicrobial. CIP has been included in the Strategic National Stockpile, which is maintained by the U.S. Department of Health and Human Services, to control bacterial infection during a national emergency such as a nuclear detonation or other radiological incident. Besides the antimicrobial activity, several groups reported immunomodulatory effects that CIP exerts in rodent models and human clinical trials [5], [6], improving a wide spectrum of conditions including thrombocytopenia [7], [8], [9], Crohn’s disease [10], [11], rheumatoid arthritis [12], [13] and chemotherapy-induced neutropenia [14]. These favorable improvements are irrelevant to its antimicrobial activity, but rather are ascribed to two general immunomodulatory actions that FQs may share: stimulation of hematopoiesis by enhanced IL-3 and GM-CSF production [15], [16] and reduction of inflammation mediated by IL-1, IL-6, and TNF-α [5]. However, whether CIP, through these immunomodulatory effects, could improve survival after CI was not studied.

Here we are the first to report that CIP modulates pathophysiological changes after CI. CIP significantly reduced levels of pro-inflammatory cytokines, while it potentiated IL-3 production, maintained GM-CSF production, and enhanced bone marrow repopulation. In the ilea of surviving mice, CIP limited apoptosis and autophagy, which may have prevented systemic infection from bacterial translocation. These CIP-mediated changes may have contributed to improve 10-day survivability in CI-mice compared to vehicle-treated control animals.

## Results

### Ciprofloxacin Increased Survival of CI-mice

**Figure 1:**
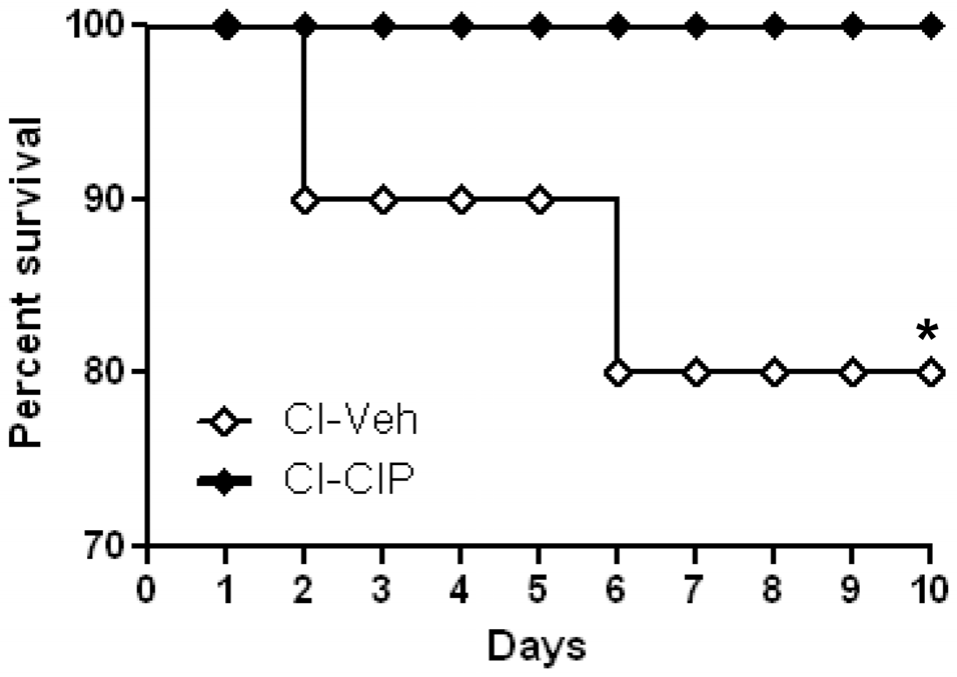
CIP improved survival after irradiation combined with skin-wound trauma (CI). Mice received 9.75 Gy ^60^Co γ-photon radiation followed within 1 h by 15% TBSA skin wound (CI). Mice were given CIP (90 mg/kg *p.o. q.d.*) or vehicle for 11 days starting day 0≤2 h after CI. Experiment was repeated to achieve statistical significance. n = 10 per group, *p<0.05 vs. CI+CIP. CI: combined injury; CIP: ciprofloxacin; Veh: vehicle.

After CI, none of the CIP-treated mice died but two vehicle-treated mice died on days 2 and 6, respectively (Figure 1). Neither RI nor wound was applied to control mice (Sham), and no animal died in such groups regardless of the treatments (data not shown). In B6D2F1/J mice LD_50/30_ for CI is 8.95 Gy, and, therefore, further mortality would be expected after day 10, as described previously [3]. We concluded that it is critical to analyze pathophysiological changes seen at day 10 that would directly link to ongoing mortality, which CIP had ameliorated.

### Ciprofloxacin Modulated Cytokine and Chemokine Concentrations in Sera of CI-mice

**Figure 2:**
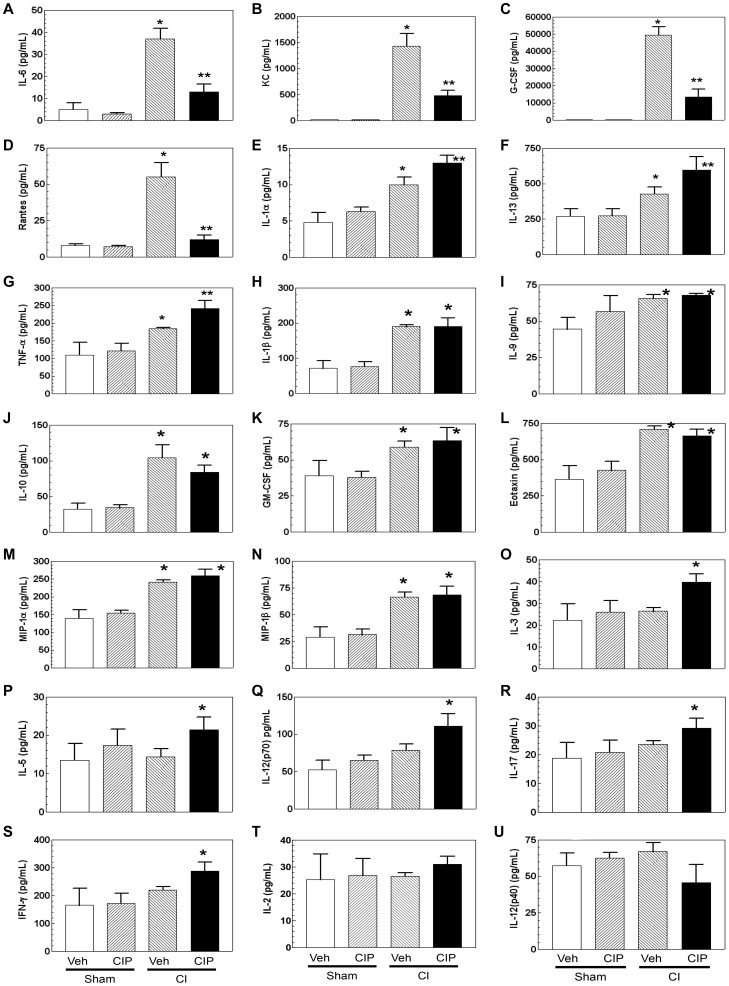
CIP altered cytokine and chemokine profiles in serum of CI-mice. On day 10 after CI, whole blood was collected from surviving mice and the sera were separated. Sera were tested for their cytokine and chemokine concentrations. n = 3−5 per group; For A-G, *p<0.05 vs. sham-Veh, sham-CIP, and CI+CIP; **p<0.05 vs. sham-Veh, sham-CIP, and CI-Veh; for H-N, *p<0.05 vs. sham-Veh and sham-CIP; for O-S, *p<0.05 vs. sham-Veh, sham-CIP, and CI-Veh. CI: combined injury; CIP: ciprofloxacin; Veh: vehicle.

Because CI could alter concentrations of cytokines and chemokines in serum [3], [4], effects of CIP on CI-induced alteration of cytokine/chemokine profiles were studied. We measured cytokines in sera of CI-mice on day 10. IL-6, KC, G-CSF, Rantes, IL-1α, IL-13, TNF-α, IL-1β, IL-9, IL-10, GM-CSF, eotaxin and MIP-1α/β increased in CI-mice. The increases in IL-6, KC, G-CSF, IL-13, IL-1β, IL-9, IL-10, eotaxin and MIP-1α/β were consistent with observations on day 7 after CI in the previous report, in which the experiment was performed under the same conditions [3]. CIP treatment reduced IL-6, KC, G-CSF and Rantes, which were significantly lower than in vehicle-treated CI-mice (Figure 2A–D). CIP further increased IL-1α, IL-13 and TNF-α to significantly higher concentrations in CI-mice than in vehicle-treated mice (Figure 2E–G); but CIP did not further increase IL-1β, IL-9, IL-10, GM-CSF, eotaxin and MIP-1α/β in CI-mice (Figure 2H–N). Although CI did not significantly change IL-3, IL-5, IL-12(p70), IL-17, and IFN-γ, CIP treatment increased their concentrations only in CI-mice significantly above vehicle (Figure 2O–S). Neither CI nor CIP had modulating effect on IL-2 or IL-12(p40) concentrations (Figure 2T–U).

### Ciprofloxacin Failed to Improve WBC and Platelet Depletion after CI

**Figure 3:**
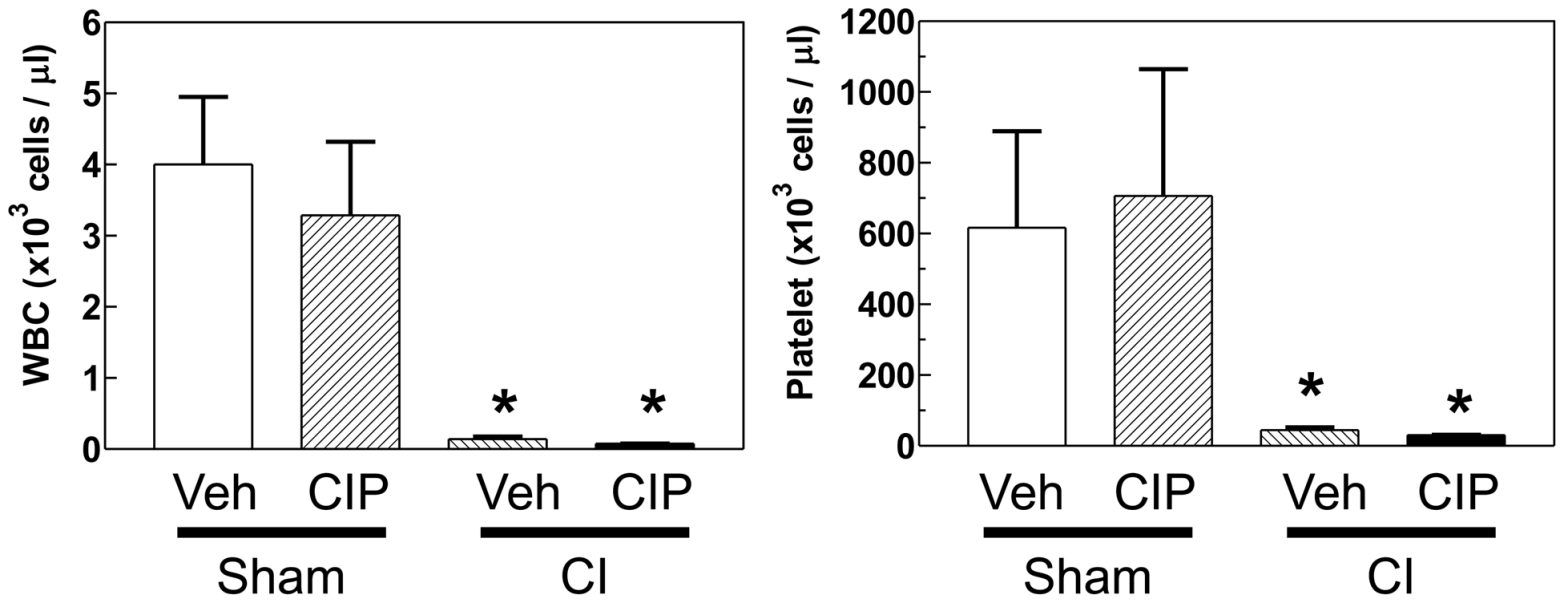
CIP did not ameliorate CI-induced depletion of white blood cells (WBC) and platelets. On day 10 after CI, whole blood was collected from surviving mice and tested for numbers of WBCs and platelets. n = 4−5 per group; *p<0.05 vs. sham-Veh, sham-CIP. CI: combined injury; CIP: ciprofloxacin; Veh: vehicle.

CI induced WBC and platelet depletion [4]. Therefore, CIP was tested for this CI-induced depletion. We did not find recovery in numbers of WBCs and platelets in CI-mice treated with CIP at this time point (Figure 3).

### Ciprofloxacin Alleviated CI-induced Bone Marrow Damage

**Figure 4:**
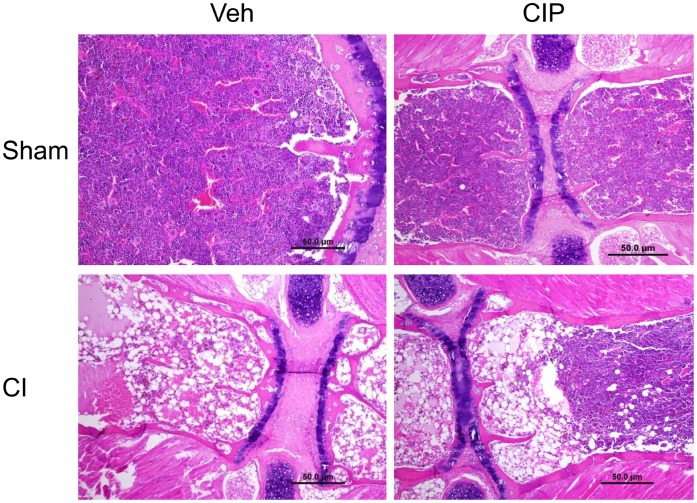
CIP accelerated bone marrow recovery after CI. On day 10 after CI, femurs were collected from surviving mice and prepared for hematoxylin-eosin staining on paraffin sections. Representative histopathology photos are shown. n = 4−5 per group. CI: combined injury; CIP: ciprofloxacin; Veh: vehicle.

Bone marrow is very sensitive to irradiation because it contains proliferative hematopoietic stem cells, whose loss would result in severe hematologic decline including WBCs and platelets (Figure 3). We observed previously the incomplete repopulation of bone marrow 30 days after CI (Fukumoto unpublished data), indicating that full repopulation requires longer than 30 days. We examined the effect of CIP on bone marrow in day-10 survivors. In vehicle-treated CI-mice, we found severe depletion of bone marrow cells demonstrated by H&E staining, which were replaced by adipocytes. In contrast, bone marrows of CIP-treated animals retained more cells resulting in lesser such replacement (Figure 4). We did not find any loss of bone marrow cells in sham-operated control mice regardless of CIP treatment (Figure 4). The sections evaluated consisted of slides made of the entire femur of the mouse so as to maintain the architecture of the marrow elements and have repeatable microscopic fields for evaluation. Thus, significantly better-populated bone marrow in CIP-treated animals may have improved hematologic parameters later than day 10 after CI.

### Ciprofloxacin Improved CI-induced Ileum Damage

**Figure 5:**
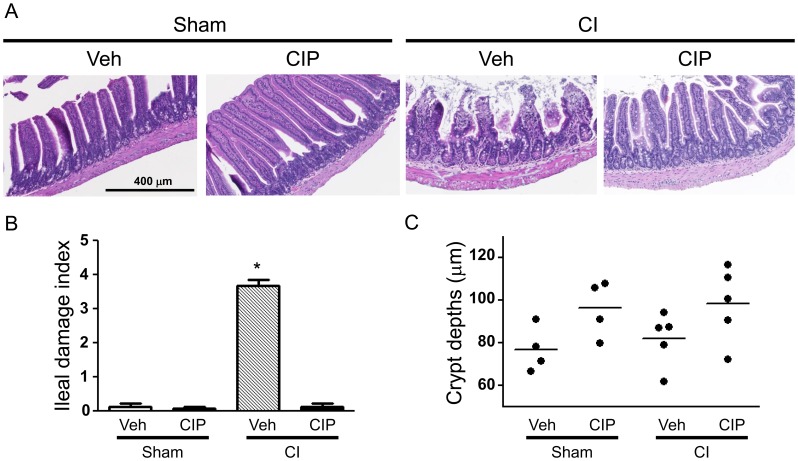
CIP inhibited ileum injury after CI. On day 10 after CI, ileal samples were collected from surviving mice and prepared for hematoxylin-eosin staining on paraffin sections. (A) Representative histopathology photos are shown, (B) An average mucosal injury grade was calculated as a damage index of 5 villa in 3 representative mice (*n* = 15) per group. *p<0.05 vs. sham-Veh, sham-CIP, and CI+CIP, and (C) Crypt depth was measured. *n* = 4−5 per group. CI: combined injury; CIP: ciprofloxacin; Veh: vehicle.

Previous studies showed that CI induced defects in intestinal wall, through which bacteria might have penetrated to cause systemic infection after CI [3]. We first assessed the damage levels of ileal villi and crypts 10 days after CI (Figure 5), when sepsis began to contribute to foreseen death [3]. We found healthy ileal mucosa in sham-treated animals regardless of CIP treatment (Figure 5A). On the other hand, in CI-mice treated with vehicle, significant mucosal injury was observed by the number of shortened villi (Figure 5A) and index of severity of damage (Figure 5B) but not the depth of crypts (Figure 5C). CIP treatment significantly improved the ileal villus structure by reducing the ileal mucosal damage (Figure 5A, B). There was a trend of moderate increase in crypt depths after CIP treatment (Figure 5C).

**Figure 6:**
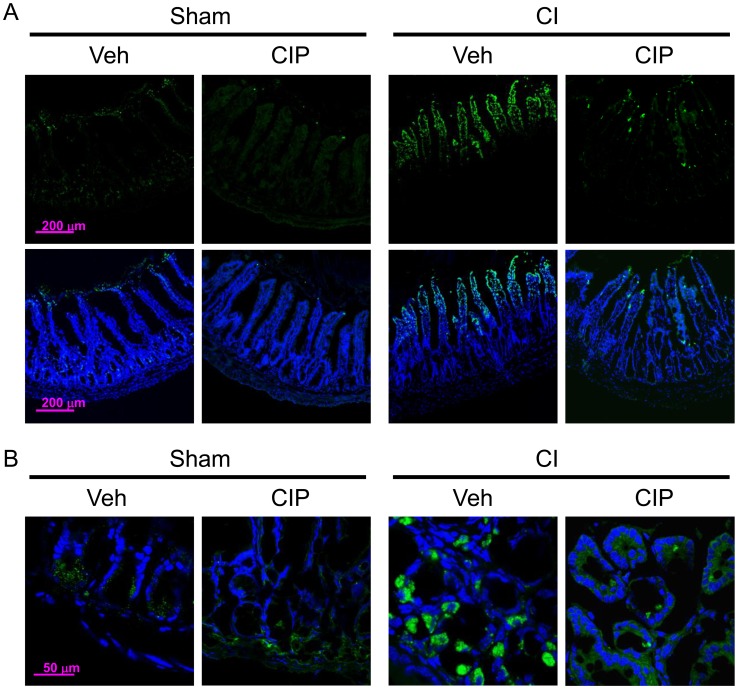
CIP inhibited apoptosis and autophagy in ileum after CI. On day 10 after CI, ileal samples were collected from surviving mice and prepared for immunofluorescent staining on frozen sections. (A) TUNEL assay was performed to identify apoptotic population (green) and presented with (bottom) or without (top) nucleus (blue), and (B) LC3 was stained to identify autophagic population and presented with nucleus (blue). Representative pictures are shown. n = 4−5 per group. CI: combined injury; CIP: ciprofloxacin; Veh: vehicle.

CIP evidently prevents and corrects the loss of ileal villi after CI. This action may be due to the adjustment in the process of cell death by apoptosis (type I death) [17] or autophagy (type II death) [18] or necroptosis (type III) [19], [20]. We determined the numbers of cells existing in ilea of survivors on day 10 by immunofluorescent staining for distinguishing apoptotic and autophagic markers and confocal microscopic imaging (Figure 6). For this analysis, some of few intact ileal villi of CI-vehicle-treated mice were chosen for the comparison with CIP-treated specimens. Using TUNEL assay, we found that CI-induced apoptosis appeared mainly in villous epithelial cells, but also in inside villi where capillary and lacteal locate (Figure 6A; green). Remarkably, CIP treatment significantly reduced numbers of apoptotic cells. CIP treatment also significantly reduced the number of cells that underwent CI-induced autophagy indicated by the LC3 presence in crypt cells (Figure 6B; green). In contrast to apoptosis seen in villous epithelium, autophagic cells were found only in crypt cells as previously described [18].

### Ciprofloxacin Decreased CI-induced Caspase-3 Activation

**Figure 7:**
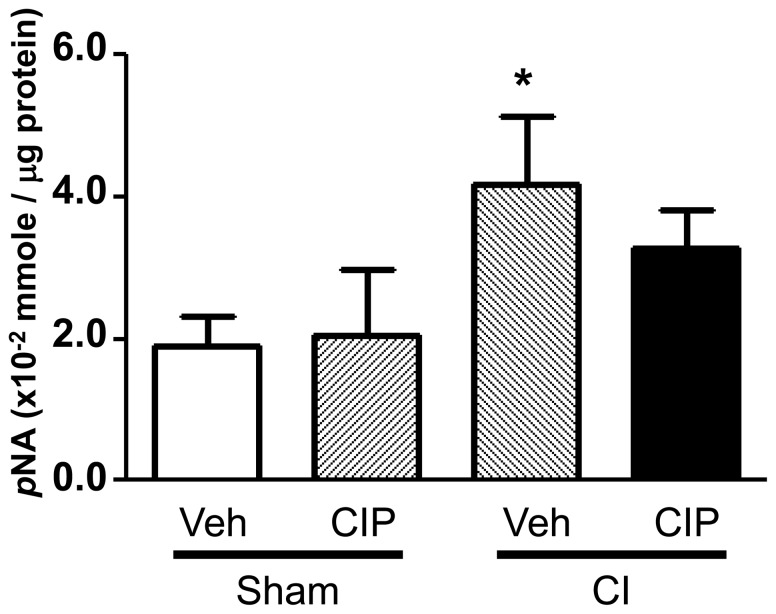
CIP inhibited the CI-induced increase in caspase-3 after CI. On day 1 after CI, ileal samples were collected from surviving mice and total cell lysates were prepared for detection of caspase-3 activity. Data is presented by the levels equivalent to *p*-nitroaniline (*p*NA). n = 6 per group; *p<0.05 vs. sham-Veh, sham-CIP, and CI+CIP. CI: combined injury; CIP: ciprofloxacin; Veh: vehicle.

To confirm ongoing apoptosis, we also measured the activity of caspase-3, a biomarker for apoptosis [21], [22], [23], in the minced lysates obtained from ilea. We chose day 1 samples obtained in a separate experiment, as it has been reported that direct radiation injury induces signaling molecules in the first phase of apoptosis, which triggers the second phase observed in day 10 samples (Figure 6A) [3]. As expected, CI induced an approximate 2-fold increase in caspase-3 activity and CIP decreased it (Figure 7). Relatively low level of induction by CI may have been due to the effect of mixed cellular population present in the lysates, as apoptosis at this time is highly specific to crypt cells and also in gut-associated lymphoid tissue (GALT), that are proliferating and sensitive to radiation [24], [25], [26]. It was suggested that the apoptosis observed in Figure 6A was a consequence of this caspase-3 activation.

### Ciprofloxacin Reduced CI-induced NF-κB Activation and Systemic Bacterial Infection

**Figure 8:**
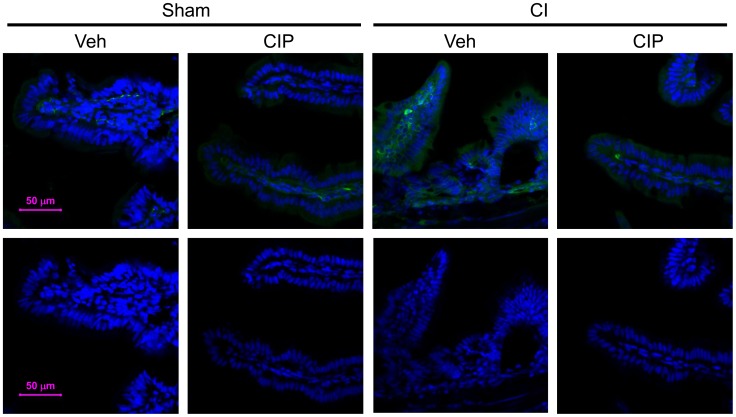
CIP inhibited the CI-induced increase in RelA expression after CI. On day 10 after CI, ileal samples were collected from surviving mice and prepared for immunofluorescent staining on frozen sections. RelA expression was detected (green) and presented with nucleus (blue). Representative pictures are shown with their fluorescence intensities for green signals (bottom). n = 4−5 per group. CI: combined injury; CIP: ciprofloxacin; Veh: vehicle.

We previously reported CI-induced systemic bacterial infection that might cause activation of iNOS and subsequently lead to increased mortality. iNOS is also known to be regulated by NF-κB, a transcription factor. We, therefore, investigated the effect of CIP on the CI-induced NF-κB activation and systemic bacterial infection. We first analyzed the level of the p65 subunit of NF-κB, also known as RelA, in the ileal samples. We found that CI significantly increased RelA in ileal villi of vehicle-treated CI-mice, which CIP treatment significantly reduced (Figure 8; green). Signal was significantly present not only on epithelial cells but also seen inside villi. CIP also suppressed the RelA basal level in sham-operated mice. It was suggested that strong activation of RelA inside villi seen in effector immune cells encountered bacteria broken into intestinal barrier. Evidence supports that lipopolysaccharide (LPS)-toll-like receptor 4 (TLR4)-mediated signaling strongly induces NF-κB activation *in vivo* [27] and *ex vivo* [28].

In an independent experiment, the ventricular heart blood, liver, and spleen were collected aseptically from moribund CI-mice to culture bacteria. Bacteria were found from all of such animals. In CI-vehicle mice, five Gram-positive and two Gram-negative bacterial species were detected. Gram–positive species were found in 6 out of 7 mice and Gram–negative species were found in 5 out of 7 mice; whereas in CI-CIP-treated mice, only three Gram-positive species were found in 3 out of 3 mice. The absence of Gram-negative bacteria suggested that CIP effectively eliminated target microorganisms. Of note, the bacterial species that caused sepsis after CI were always found in the ileal lumen of the same animals when tested (data available for the names of bacterial species).

### Ciprofloxacin Inhibited CI-induced Increases in IgA

**Figure 9:**
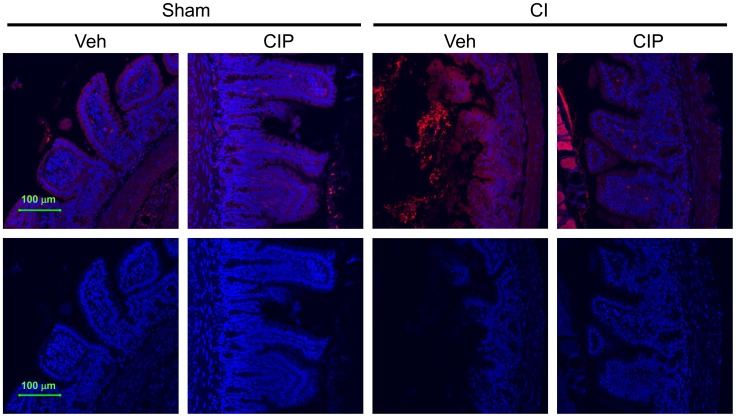
CIP inhibited the CI-induced increase in IgA expression after CI. On day 10 after CI, ileal samples were collected from surviving mice and prepared for immunofluorescent staining on paraffin sections. IgA expression was detected (red) with counterstaining with nucleus (blue). Representative pictures are shown with their fluorescence intensities for red signals (bottom). n = 4−5 per group. CI: combined injury; CIP: ciprofloxacin; Veh: vehicle.

Immunoglobulin A (IgA) is a unique antibody that is found on the intestinal mucosal surface as soluble dimers and protects it from bacterial and viral infection by this route [29]. It has also been proposed that plasma cells in lamina propria acquire the ability to produce IgA after the stimulation by inflammation mediated by IL-6 [30]. Because CI increased IL-6 approximately 7-fold (Figure 2A), IgA distribution was examined in CI-mice. We performed immunofluorescent staining using a rat antibody against mouse IgA. In the ileum of sham-treated animals, we observed uniform distribution of IgA on the mucosal surface as well as on specific cells that are present in lamina propria (Figure 9; red). After CI, the level of IgA increased at a significant level together with increased damage to villi, and CIP treatment reduced both of them. IgA levels were consistently higher on villi with damage in all specimens observed as indicated by signal intensity (Figure 9; bottom), but the changes in the number of IgA^+^ plasma cells in lamina propria did not reach statistical significance.

## Discussion

There is a pressing need to find countermeasures to treat RI and CI. At present, none of countermeasures yet evaluated to treat either condition has been approved by the U.S. FDA for the use in humans based upon evaluation in two suitable laboratory animal species. In the search for appropriate CI-treatment targets, it is necessary to understand the mechanisms underlying CI. Our research has been investigating many pathophysiological changes that occurred during the period of 30 days after CI, which identified increased DNA damage, stress-response gene expressions, serum cytokine and chemokine concentrations, ileal damage, bacterial translocation [3], and blood-cell depletion [4]. Some of these changes were truly unique to CI, *i.e.*, changes were great enough to distinguish CI from any other single injuries, and offered us multiple treatment targets or parameters to monitor prospective countermeasure development.

CIP is a second-generation fluoroquinolone (FQ) [31]. The spectrum of antimicrobial activity of CIP is due to the specificity in inhibition directed to bacterial DNA gyrase and topoisomerase IV, particularly in Gram-negative species [32]. The pharmacokinetics [33] and pharmacodynamics [34] in humans have been well documented. It is well tolerated, distributes rapidly in tissues, and has similar half-lives in mice [35], [36], [37] and humans [33], and is readily available in several formulations for oral, intravenous, intraperitoneal, and topical routes. These factors meet the needs for the management of bacterial infection in a mass-casualty scenario. Although it has been used after the anthrax attack in 2001 [38], [39], it has never been tested for a treatment of CI.

The wide use of CIP in clinical settings has helped the discovery of its immunomodulatory effect, apart from the antimicrobial activity. Since the stimulation of hematopoiesis highlights immunomodulation by CIP [15], it might combat the CI-induced severe hematologic loss. FQs including CIP were shown *ex vivo* to enhance cytokines IL-3 and GM-CSF in mouse splenocytes that gave rise to enhanced hematopoiesis [15], while CIP, together with a mitogen, induced hyper-production of IL-2 in human peripheral lymphocytes [40]. Only a subset of FQs produces this induction effect, which appears to be associated with the cyclopropyl moiety present at the N1 position [15]. Although the exact mechanism is elusive, it is possible that transcription factors, which are common to increased cytokines, such as activator protein-1 (AP-1), are activated by those FQs [41].

On the other hand, it has also been reported that FQs including CIP inhibited mammalian topoisomerase II [42]. Although this inhibitory effect is about 1,000-fold less than the one directed to bacterial counterparts, it is not negligible when considering sustained accumulation of FQs in the organs [43] and intracellularly up to 12-fold greater than extracellular concentrations [5], [44], [45]. Inhibition of topoisomerase II resulted in lowered inflammatory cytokines TNF and IL-6 secretion from *ex vivo* splenocytes, a majority of which was monocytes, and also lowered MIP-1α in macrophages *in vitro* [46]. Therefore, the topoisomerase II inhibitory effect of CIP may be a benefit in treating enhanced inflammation induced by CI.

In the present study, for the first time, we investigated whether CIP mitigated ionizing radiation combined injury (CI) through its effects on survival, cytokine levels, bone marrow cells, and ileum of surviving mice on day 10 after CI. The multiple effects of CIP were observed in several tissues. In the sera, CIP effectively reduced CI-induced pro-inflammatory cytokines IL-6, KC (*i.e.*, IL-8 in human), and G-CSF up to 80% and Rantes to basal level (Figure 2A–D). The enormous induction of IL-6, KC, and G-CSF in serum in CI-mice correlated with the results reported previously from this laboratory on day 7 [3]. Continuous, high IL-6 expression caused serious secondary damage to the organs rather than its benefits obtained by mild and transient expression [47]. It is also well known, for example, that IL-6 is very high in inflammatory bowel disease (IBD) including ulcerative colitis (UC) and Crohn’s disease (CD), and exists in foci specific manner [48], suggesting its major roles in IBD pathology. In fact, IL-6 is considered as a treatment target in IBD and other inflammatory conditions [49], [50]. Other major inflammatory cytokines such as KC and Rantes are also known to induce serious tissue damage, if their levels are too high and meet no resolution [51], [52].

IL-3 is an essential mediator for myeloid lineage development and has been reported to be elevated by CIP treatment [15]. In our study, CIP significantly increased IL-3 only in CI-animals (Figure 2O). Further, CIP also accelerated bone-marrow recovery that may be related to increased level in IL-3 (Figure 4), while the recovery in WBCs and platelets was not yet to be seen (Figure 3). IL-3 in combination with erythropoietin (EPO) has been reported to increase production of WBC [53]. Therefore, the observation of accelerated bone-marrow recovery by CIP after CI warrants future studies on the association of the IL-3, EPO and bone marrow recovery with WBC production.

CIP limited physical damages occurring in the ileum after CI (Figure 5A–C). Analysis on cell-death type-specific markers revealed that after CI villous epithelial cells underwent apoptosis (Figure 6A) while crypt cells displayed elevated formation of autophagosomes (Figure 6B). CIP effectively reduced both types of cell death. A high level of IgA coated the ileal mucosa of CI-mice, particularly in foci exhibiting severe morphological damage in villous tips. This may be a result of massive IL-6 concentration observed in sera, both of which CIP reduced to normal range (Figure 9). The cell death, which occurred on the villi epithelial cells, was associated with activation of NF-κB in immune effector cells inside villi and a consequence of caspase-3 earlier (Figures 7 and 8). This finding correlates with observations found in human T cells *in vitro* [54], [55] and in CD2F1 mice [56]. Similarly, the autophagic death, which occurred in crypt cells of CI-mice in our study, was also found in CD2F1 mice [18].

CIP offers several advantages to be developed further as a drug to treat CI in a mass-casualty scenario: (1) it is included in the Strategic National Stockpile for bacterial infection control; (2) it can be taken orally so that patients can self-administer it; (3) it possesses not only antimicrobial but also favorable immunomodulatory activity; and (4) it is inexpensive.

In summary, CIP significantly increased survival, altered the serum cytokine and chemokine profile, accelerated bone-marrow recovery, inhibited cell death of ileum, and prevented systemic bacterial infection. These effects could be mediated by its capability of inhibiting NF-κB and caspase-3. Therefore, the results suggest that CIP may prove to be beneficial for treating critical sequelae of CI.

## Materials and Methods

### Ethics Statement

Research was conducted in a facility accredited by the Association for Assessment and Accreditation of Laboratory Animal Care-International (AAALACI). All procedures involving animals were reviewed and approved by the AFRRI Institutional Animal Care and Use Committee. Euthanasia was carried out in accordance with the recommendations and guidelines of the American Veterinary Medical Association.

### Animals

Female B6D2F1/J mice were purchased from Jackson Laboratory (Bar Harbor, ME) and were used when 33–36 weeks old with 25–35-g average weights. Male mice were not used in this study because of problems associated with aggression, which in these experiments could lead to further damage to wound sites and enhanced infection. All mice were randomly assigned to experimental groups. No more than 4 mice were housed per filter-topped polycarbonate cage (MicroIsolator) in conventional holding rooms. Rooms were provided with 10–15 changes per hour of 100% fresh air conditioned to 72±2°F with a relative humidity of 50±20%. Mice were maintained on a 12-h light/dark, full-spectrum light cycle with no twilight. Four or five days prior to the experiments, body weights were measured and hair of the dorsal surface was removed using electric clippers. On the day of experiments, mice were first irradiated and then wounded under anesthesia by methoxyflurane inhalation. All mice, including controls, received an intraperitoneal injection of 0.5 mL sterile isotonic 0.9% NaCl as fluid therapy immediately after combined injury or sham injury to avoid radiation-induced dehydration. After injuries mice were assigned to clean cages and provided with proper food and acidified water.

### Radiation Injury (RI)

Mice were placed in well-ventilated acrylic restrainers and given 9.75 Gy of whole-body ^60^Co γ-photon radiation delivered at a dose rate of approximately 0.4 Gy/min. Dosimetry was performed using the alanine/electron paramagnetic resonance system. Calibration of the dose rate with alanine was traceable to the National Institute of Standards and Technology and the National Physics Laboratory of the United Kingdom. Sham-irradiated mice were placed in the same acrylic restrainers, taken to the radiation facility, and restrained for the time required for irradiation.

### Wound Trauma

Within 1 h of irradiation, mice were anesthetized under methoxyflurane by inhalation; an experimental wound was administered 19±1.3 mm from the occipital bone and between the scapulae using a stainless steel punch on a Teflon®-covered board cleaned with 70% alcohol before each use. The panniculus carnosus muscle and overlying skin (23.5±1.1 mm long and 14.9±0.7 mm wide) were removed. Sham-wounded mice were treated identically to other groups except without wounding.

### Preparation and Administration of Ciprofloxacin

Veterinary-, oral-use ciprofloxacin tablets (500 mg/each) (Dr. Reddy’s laboratories, Hyderabad, India) were used to prepare fresh solution every week. Tablets were ground, dissolved in sterile water (vehicle) and after a brief centrifugation sterile-filtered using 0.22 µm CN (cellulose nitrate) filter system (Corning; Corning, NY). Each dose of 0.2 mL ciprofloxacin solution delivered 90 mg/kg based on average body weight. All mice received 0.2 mL of either ciprofloxacin or vehicle via oral route once per day for 11 days, starting within 2 h of CI and through day 10. The treatment regimen has been justified based on previous studies [57], [58] and pharmacokinetics data [59] to treat more severe polymicrobial endogenous sepsis in CI [3]. Mice were gently restrained by hand and fed using oral feeding needles attached to 1-mL syringes. Feeding needles were disinfected by 70% ethanol on a gauze sponge between mice in each cage and new needles were used for every cage of mice.

### Survival

Survival after CI and therapy with CIP was evaluated with 10 mice per group. The gross appearance, general health, and survival of each mouse were followed by visual inspection daily for 10 days in parallel with other assessments.

### Histopathology Assessment

Ileal and femur specimens were collected for histopathology 10 days after CI (n = 4−5 per group). Specimens were immediately fixed in 10% phosphate-buffered formalin upon removal. The tissue was then embedded in paraffin, sectioned transversely and stained with hematoxylin and eosin (H&E). Tissue imaging and analysis were performed by the NanoZoomer 2.0 from HAMAMATSU PHOTONICS K.K. (Hamamatsu, Japan). The same acquisition setting, including scaling, applies to all images in the same figure. The mucosal damage of ileum for each slide was graded on a six-tiered index defined by Chiu et al. [60], [61] as follows: grade 0, normal mucosa; grade 1, development of subepithelial spaces near the tips of the villi with capillary congestion; grade 2, extension of the subepithelial space with moderate epithelial lifting from the lamina propria; grade 3, significant epithelial lifting along the length of the villi with a few denuded villus tips; grade 4, denuded villi with exposed lamina propria and dilated capillaries; and grade 5, disintegration of the lamina propria, hemorrhage, and ulceration. The bone marrow cellularity was assessed by a board certified veterinary pathologist in a blinded manner through the averaging of total erythroid and myeloid cells over 10 high-powered fields, subsequent to a low power qualitative assessment. The megakaryocytes were also counted and averaged over 10 high-powered fields. Since regenerative foci can be unbalanced within a section, they were included in the measurement over 10 fields based on the low power overview of the whole section.

### Antibodies

The following antibodies were used for the analyses by immunofluorescent staining: mouse monoclonal to NF-κB p65 (F-6) and goat polyclonal to MAP LC3 (Santa Cruz Biotechnology, Inc.; Santa Cruz, CA); rat monoclonal anti‐mouse IgA purified (eBioscience; San Diego, CA) provided by Ms. Kristen Gambles; Alexa Fluor® 488 goat anti-mouse IgG and Alexa Fluor® 568 goat anti-rat IgG (Life Technologies Corporation; Grand Island, NY).

### Immunofluorescent Staining

Ileal specimens were collected for immunofluorescent antibody staining 10 days after CI (n = 8−10 per group). Specimens were immediately fixed in phosphate-buffered 4% paraformaldehyde (FD NeuroTechnologies, Inc.; Baltimore, MD) at 4°C for overnight. They were then washed twice by ice-cold phosphate-buffered saline (PBS) before and after treatment with 20% sucrose in PBS at 4°C for 2 h. Resulting tissues were dried briefly on paper towels and embedded in Tissue-Tek® O.C.T. compound (Sakura Finetek USA, Inc.; Torrance, CA) on dry-ice. Tissues were kept frozen at −80°C until sectioning on cryostat and used for immunofluorescent staining. In some studies, paraffin sections were also used for the staining as noted. Prepared slides were treated with Target Retrieval Solution and Protein Block Serum-Free (Dako North America, Inc.; Carpinteria, CA) according to the manufacturer’s protocol, and stained with respective primary and secondary antibodies with washing between and after with PBS with 0.1% Tween® 20. Resulting slides were briefly washed with PBS and desalted by soaking in distilled-deionized water and sealed by coverslips in mounting medium with DAPI (Life Technologies Corporation). Terminal deoxynucleotidyl transferase biotin–dUTP nick end labelling (TUNEL) staining and the following image processing were conducted with a provision of the manufacturer’s recommendations (EMD Millipore Corporation; Billerica, MA) [18].

### Microimaging Analysis

A Zeiss LSM710 laser scanning confocal microscope (Carl Zeiss MicroImaging; Thornwood, NY) with EC Plan-Neofluar 10×/0.3, Plan-Apochromat 20×/0.8, and EC Plan-Neofluar 40×/0.75 objectives were used to scan the signals. Intensity of signals were also measured and shown as noted. The same acquisition setting, including scaling, applies to all images in the same figure.

### Cytokine and Chemokine Measurements

Whole blood (0.7–1.0 mL) was collected by terminal cardiac puncture from mice anesthetized by methoxyflurane 10 days after CI. CapiJect tubes (Terumo; Somerset, NJ) were used to separate sera by centrifugation at 3,500 g for 90 seconds and stored at −70°C until assayed (n = 3−5 per group). Cytokine concentrations were analyzed using the Bio-Plex™ Cytokine Assay (Bio-Rad; Hercules, CA) following the manufacturer’s directions. Briefly, serum from each animal was diluted fourfold and examined in duplicate. Data were analyzed using the LuminexH 100™ System (Luminex Corp.; Austin, TX) and quantified using MiraiBio MasterPlexH CT and QT Software (Hitachi Software Engineering America Ltd.; San Francisco, CA), and concentrations were expressed in pg/mL unless otherwise noted. The cytokines analyzed were interleukin (IL)-1α, IL-1β, IL-2, IL-3, IL-4, IL-5, IL-6, IL-9, IL-10, IL-12(p40), IL-12(p70), IL-13, IL-17, eotaxin, G-CSF, GM-CSF, IFN-γ, KC (*i.e.*, IL-8 in human), MCP-1, MIP-1α, MIP-1β, RANTES and TNF-α.

### Caspase-3 Measurement

Caspase-3 activity was determined using the Caspase-3 Colorimetric Activity Assay Kit, DEVD, (EMD Millipore Corporation). In brief, 10 µl of each sample lysate was added to wells of a 96-well plate already containing the substrate Ac-DEVD-*p*-nitroaniline (Ac-DEVD-*p*NA). Caspase in the lysates cleaved the substrate to form *p*NA as indicated by an increase in absorbance at 405 nm, which was measured using a SpectraMax 250 spectrophotometric plate reader and SOFTmax Pro 3.1.1 software (Molecular Devices; San Diego, CA). Data were normalized to total protein contents, and capase-3 protein was expressed as pmole *p*NA/µg protein.

### Detection of Bacterial Translocation

The detailed methods used for detecting bacteria in blood and tissues to assess bacterial translocation were previously described [3]. Briefly, mice were dissected aseptically to isolate facultative bacteria from selected tissues. The apex of the heart was cut and the cut surface was immediately applied directly to 5% Sheep Blood Agar (SBA), Colistin-Nalidixic Acid in 5% Sheep Blood Agar (CNA), and Xylose-lysine-desoxycholate agar (XLD) media (Remel Microbiology Products; Lenexa, Kansas). Samples of liver, spleen and ileum were removed and homogenized by crushing with a sterile cotton or polyester swab in a sterile Petri dish and inoculated immediately onto SBA, CNA, and XLD media. SBA and CNA were incubated in 5% CO_2_ at 35°C for 18–24 h, XLD plates were incubated at 35°C. Single colonies of isolated microorganisms were Gram-stained and identified by a Vitek2 Compact automated system (bioMérieux, Inc.; Durham, NC).

### Statistical Analysis

Five mice per group were used for each survival experiment, and it was repeated to gain statistical significance by Mantel-Cox procedure (total n = 10/group). All other results are expressed as means ± SEM. One-way ANOVA, two-way ANOVA, studentized-range test, and χ^2^ test were used for comparison of groups; 5% was used as the level of significance.
